# Hsa_circ_0123190 acts as a competitive endogenous RNA to regulate APLNR expression by sponging hsa-miR-483-3p in lupus nephritis

**DOI:** 10.1186/s13075-020-02404-8

**Published:** 2021-01-13

**Authors:** Chunyi Zhang, Congcong Gao, Xueqi Di, Siwan Cui, Wenfang Liang, Wenbo Sun, Menghui Yao, Shengyun Liu, Zhaohui Zheng

**Affiliations:** Department of Rheumatology, The First Affiliated Hospital of Zhengzhou University, 1 Eastern Jianshe Road, Zhengzhou, 450052 Henan China

**Keywords:** circRNAs, MicroRNAs, Competitive endogenous RNAs, Lupus nephritis, Systemic lupus erythematosus

## Abstract

**Background:**

Lupus nephritis (LN) is one of the most severe complications of systemic lupus erythematosus (SLE). Circular RNAs (circRNAs) can act as competitive endogenous RNAs (ceRNAs) to regulate gene transcription, which is involved in mechanism of many diseases. However, the role of circRNA in lupus nephritis has been rarely reported. In this study, we aim to investigate the clinical value of circRNAs and explore the mechanism of circRNA involvement in the pathogenesis of LN.

**Methods:**

Renal tissues from three untreated LN patients and three normal controls (NCs) were used to identify differently expressed circRNAs by next-generation sequencing (NGS). Validated assays were used by quantitative reverse transcription polymerase chain reaction (qRT-PCR). The interactions between circRNA and miRNA, or miRNA and mRNA were further determined by luciferase reporter assay. The extent of renal fibrosis between the two groups was assessed by Masson-trichome staining and immunohistochemistry (IHC) staining.

**Results:**

159 circRNAs were significantly dysregulated in LN patients compared with NCs. The expression of hsa_circ_0123190 was significantly decreased in the renal tissues of patients with LN (*P* = 0.014). Bio-informatics analysis and luciferase reporter assay illustrated that hsa_circ_0123190 can act as a sponge for hsa-miR-483-3p, which was also validated to interact with APLNR. APLNR mRNA expression was related with chronicity index (CI) of LN (*P* = 0.033, *R*^2^ = 0.452). Moreover, the fibrotic-related protein, transforming growth factor-β1 (TGF-β1), which was regulated by APLNR, was more pronounced in the LN group (*P* = 0.018).

**Conclusion:**

Hsa_circ_0123190 may function as a ceRNA to regulate APLNR expression by sponging hsa-miR-483-3p in LN.

## Background

Lupus nephritis (LN) is one of the most common and severe complications of systemic lupus erythematosus (SLE) and seriously affects the quality of life and prognosis of SLE patients. About 80% of children and 60% of adults are susceptible, and up to 30% of patients could progress to end-stage renal disease (ESRD) [1]. Previous studies investigated 491 cases of LN patients in central plains of China with 5-year, 10-year, 15-year, and 20-year survival rates of 88%, 77%, 53%, and 46%, respectively, and found that renal failure was the main cause of death [2]. The loss of labor in patients with LN who have advanced to end-stage renal disease places an enormous economic burden on society and families. Therefore, exploring the pathogenesis of LN is important and urgent.

Circular RNA (circRNA) is a new type of non-coding RNA, which produced from precursor mRNA back-splicing by covalently closed, single-stranded RNA circles at the junction site of 3’5’-phosphodiester bond [3]. CircRNA expression is more stable to be detected because of its closed circular structure. Due to the cell type-specific or tissue-specific manner [4, 5], circRNAs are involved in the pathogenesis of various human diseases. CircRNAs are rich in microRNA binding sites, acting as miRNA sponges and regulating gene transcription, which is called competitive endogenous RNA (ceRNA) molecules [6, 7]. In the studies on renal diseases, Wang et al. reported that androgen receptor enhanced migration and invasion of renal transparent cell carcinoma by inhibiting the expression of circHTAT1 regulating miR-195-5p/29a-3p/29c-3p [8]. In addition, circRNA ZNF609 was found to regulate fork head box P4 (FOXP4) expression by targeting miR-138-5p in renal carcinoma [9]. However, the role and mechanism of circRNAs in LN has been rarely reported.

Renal fibrosis is a common pathological feature of progressive LN, which is closely related to ESRD [10, 11]. Apelin and its receptor (apelin receptor, APLNR) can be widely distributed in the heart, lung, pancreas, kidney, and other tissues [12]. Numerous evidences indicate that apelin and APLNR play a key role in various kidney diseases, such as renal fibrosis, renal ischemia/reperfusion injury, polycystic kidney disease, and diabetic nephropathy [13]. Particularly, apelin and APLNR could inhibit the deposition of extracellular matrix (ECM) and attenuate renal fibrosis by acting on TGF-β [14]. In the present study, we firstly established the circRNA expression profile in the kidney tissues of patients with LN. And then, we further explored that hsa_circ_0123190 was a novel biomarker of peripheral blood for LN and could act as a sponge for hsa-miR-483-3p to regulate APLNR expression involved in renal fibrosis in LN.

## Methods

### Subjects and samples

A total of 10 LN patients with renal biopsy were enrolled in this study between May 2018 and December 2018 from the Department of Rheumatology and Immunology of the First Affiliated Hospital of Zhengzhou University. Five patients with renal tumor were from the Urology Department at the same hospital. Ten peripheral blood samples were collected from the volunteers as healthy controls (HCs). All patients and volunteers were female, between 18 and 60 years old. The following were exclusion criteria: (1) patients with serious infection within 1 month before admission, (2) patients with malignant tumors, (3) patients with other autoimmune diseases, (4) patients with pregnancy, and (5) patients with eGFR lower than 30 mL/min/1.73 m^2^.

Kidney tissues from LN patients were obtained from renal biopsies before the treatment with steroid and/or immunosuppressant. Renal normal controls (NCs) were kidney tissues at least 5 cm from the edge of tumor from patients with renal cancer, and then confirmed to be normal histological morphology under microscopy. All fresh tissues were stored in RNAlater® Solution (Thermo Fisher Scientific, CA, USA) and then frozen in − 80 °C until RNA extraction. All peripheral blood samples (2 mL) were drawn from the median cubital vein with a PAXgene Blood RNA Tube (Qiagen, Hilden, Germany).

### NGS profiling analysis

Total RNA was extracted from the frozen renal tissues with using Trizol LS reagent (Invitrogen, CA, USA). Total RNA from fresh peripheral blood samples is isolated by PAXgene Blood RNA Kit (Qiagen, Hilden, Germany). Total RNA was quantified and qualified by an Agilent 2100 Bioanalyzer (Agilent Technologies, CA, USA), NanoDrop™ 2000 spectrophotometer (Thermo Fisher Scientific, CA, USA) and 1% agarose gel. The criteria of total RNA was used for subsequent library preparation: (1) the value of OD_260/280_ was between1.8 ~ 2.2 and OD_260/230_ was above 2.0, and (2) the value of RIN was above seven.

NGS library preparation was performed by the Genesky Biotechnologies Inc. (Shanghai, China). The rRNA was depleted from total RNA by Ribo-Zero^TM^ rRNA removal kit (Human/Mouse/Rat) (Illumina, CA, USA) before building the RNA-seq library. After purification, divalent cations at higher temperatures were applied for making small pieces of fragments of the residual RNA fractions. The reverse transcription of all the cleaved RNA fragments was used to construct the complementary DNA (cDNA) library with TruSeq Stranded Total RNA Library Prep Kit (Illumina, CA, USA) according to the manufacturer’s instructions. The library quality was evaluated with Agilent 2100 Bioanalyzer (Agilent Technologies, CA, USA). RNA libraries were denatured as single-stranded DNA molecules. Finally, sequencing was carried out using a 2 × 150 base paired-end configuration with Illumina Hiseq 2500 (Illumina, CA, USA).

### GO and KEGG pathway analysis

The predicted functions of the differentially expressed circRNAs between LN and NC were conducted by gene ontology (GO, http://geneontology.org) and Kyoto Encyclopedia of Genes and Genomes (KEGG, http://www.kegg.jp) analysis. Hierarchical clustering of the differentially expressed circRNA according to three categories, the biological process (BP), cellular component (CC), and molecular function (MF) was used by GO analysis. Pathway analysis of circRNA was performed by KEGG database.

### QRT-PCR validation

The candidate circRNAs were selected according to the following: Firstly, the top 20 differently expressed circRNAs between the LN group and the control group were selected. Secondly, according to the results of GO and KEGG enrichment analysis and literature, the circRNAs with most likelihood to be related to the pathogenesis of LN was analyzed. Thirdly, the primers of circRNAs for qRT-PCR could be designed. Finally, every of the candidate circRNAs had only one circBase ID. QRT-PCR with SYBR green analysis was used to validate the expression of the selected circRNAs from circRNA profiles. Total RNA was extracted by using Trizol reagent (Invitrogen, Carlsbad, CA, USA). The cDNA was synthesized by Reverse Transcriptase M-MLV (Takara, Tokyo, Japan). In addition, qRT-PCR was performed with SYBR® Premix Ex Taq™ II (Takara, Tokyo, Japan). GAPDH was used as endogenous control for circRNA qRT-PCR and U6 for miRNA. One microgram of RNA was mixed with 1 μL of RNase R (20 U/μL) at 37 °C for 20 min and then washed with RNeasy cleaning agent. All of the primers are listed in Table 1. The relative circRNA expression was calculated using 2^−ΔΔCt^, with ΔCt = Ct _target_ – Ct _β-actin_, −ΔΔCt = − (ΔCt _sample_ − ΔCt _control_).

**Table 1:** The sequences of primers

Gene	Forward primer sequence	Reverse primer sequence
hsa_circ_0123190	TGAGGATGGAGAACCCACCAA	CCCCCATCACATGAGCACAA
hsa_circ_0000660	TGCTTCCAGTGGGAATCCACAT	TCAGAGAGCCGTAGGTTGCGTAT
hsa_circ_0007379	TCTCTTTCTCCAAGGAGCTCCACA	TGCTGATGAAGCTGAGCAGGGA
hsa_circ_0003302	TGGATGTTCCACAGGAAGAAGTGC	GGGCCACGGCGATAAGGAAAAT
APLNR	AGGCAGCAGGGCTGATGAATGG	TGCAGACACCCCTCCATCCTCT
hsa-miR-483-3p	TCACTCCTCTCCTCCCGTCTT	

### Dual-luciferase reporter assay

Luciferase reporter assays were used to detect the direct binding between selected RNAs. The hsa_circ_0123190 wild-type (WT) 3′-UTR, APLNR WT 3′-UTR and mutant sequence hsa_circ_0123190 (MUT) 3′-UTR, APLNR MUT 3′-UTR were constructed. Subsequently, we inoculated HEK293T cells into 96-well plates and co-transfected 5 pmol hsa-miR-483-3p mimics or negative control with the 0.16 μg hsa_circ_0123190 and APLNR wild-type or mutant type plasmids. We tested the fluorescence intensity using the dual-luciferase reporter gene assay system (Promega, Madison, USA) after 48 h of transfection.

### Histology and immunostaining

Paraffin-embedded kidney tissues from the human were cut at 2-μm thickness, deparaffinized, and rehydrated. Then, for histological examination, kidney sections were stained with Masson-trichrome reagent to explore the degree of fibrosis. Moreover, for immunohistochemistry (IHC) staining, kidney sections were incubated in citrate buffer for 20 min at 95 °C to retrieve antigen. Non-specific binding was blocked with 10% normal goat serum for 30 min at RT. The slides were incubated with antibodies of TGF-β1, α-smooth muscle antibody (α-SMA) overnight at 4 °C, followed by incubation with biotin-conjugated goat anti-mouse/rabbit immunoglobulin IgG for 30 min at RT, and then reacted with streptavidin-conjugated peroxidase for 30 min at RT. On IHC staining, we semi-quantified the stained slides using a specific immunohistochemical histological score technique, H-score [15]. The H-score was obtained by multiplying the staining intensity by a constant to adjust the mean to the strongest staining, to produce a score in the range of 0–300. Specifically, H-score = scale × percentage of strong staining (0–100%). H-score = 1.0 indicated a weak percentage, 2.0 indicated a moderate percentage, and 3.0 indicated a strong percentage.

### Western blot

RIPA buffer was used for tissues lysis, according to the manufacturer’s instructions, and the protein concentration was determined using a Bicinchoninic Acid (BCA) Protein Assay Kit. Proteins were separated by SDS-PAGE using Tris-Glycine 10% polyacrylamide gels in SDS page running buffer and transferred to PVDF membrane (Servicebio, Wuhan, China). The membranes were immunoblotted with antibodies against APLNR (1:500, Proteintech, 20341-1-AP) after blocking in 5% milk. Following primary antibody incubation, the membranes were probed with HRP-conjugated mouse anti-rabbit secondary antibody (1:5000, Santa Cruz, sc-374015) and imaged using the Chemidoc system (BioRad).

### Statistical analysis

Statistical software SPSS 25.0 (IBM Corporation, USA) was used for statistical analysis. Scatter diagrams were drawn by GraphPad Prism version 6.0 (GraphPad Software, Inc., CA, USA). Differences between two groups were analyzed for statistical significance by *t* test. The correlation between circRNAs level and clinical parameters of LN were analyzed by Pearson’s linear correlation. ROC curves were performed, and the specificity and sensitivity of predictive power were assessed by area under curve (AUC). A *P* value < 0.05 was considered as statistically significant.

## Results

### Profiling and characteristics of circRNAs in the renal tissues of LN patients

Firstly, we analyzed the circRNAs profiling in the renal tissues of three LN patients and three NCs by next-generation sequencing. The clinical information for the 3 patients and NCs for circRNA profiling were shown in Supplement Tables 1 and 2. Results showed that total 159 circRNAs were identified to be abnormally expressed in renal tissues of LN patients compared with NCs (log_2_|fold change| ≥ 1, *P* < 0.05), of which 73 circRNAs were significantly upregulated and 86 circRNAs were remarkably downregulated. The top 10 upregulated and downregulated circRNAs were summarized in Table 2. Hierarchical clustering heatmap and volcano plots were used to show the different expression levels of circRNAs between LN and NC (Additional file 1: Supplement Figure 1).

**Table 2:** The top 10 abnormally expressed circRNAs

circBase ID	circRNA ID	*P* value	Type
NA	chr2:153431650-153437563:+	0.038640885	Up
hsa_circ_0008683	chr14:102486230-102489217:+	0.0035304	Up
hsa_circ_0002980	chr7:141336760-141349133:+	0.004624716	Up
hsa_circ_0002153	chrX:107083900-107097934:+	0.005655858	Up
hsa_circ_0000734	chr17:1746097-1756483:+	0.040425419	Up
hsa_circ_0003757	chr1:24140680-24147083:-	0.039814259	Up
hsa_circ_0004958	chr17:58711214-58725443:+	0.039891741	Up
hsa_circ_0002319	chr3:197592983-197598333:+	0.028417612	Up
NA	chr1:246890194-246903603:+	0.032809392	Up
hsa_circ_0003302	chr11:120916383-120930794:+	0.031378282	Up
NA	chr2:40655613-40673788:-	0.014846209	Down
NA	chr4:122411146-122446439:+	0.009160292	Down
hsa_circ_0005806	chr16:30675536-30677862:+	0.004353445	Down
NA	chr4:166960491-166999182:+	0.015788499	Down
hsa_circ_0004284	chr1:71304482-71320883:+	0.002193657	Down
hsa_circ_0003602	chr3:47702784-47719801:-	0.005016822	Down
hsa_circ_0000994	chr2:40655613-40657444:-	0.027239613	Down
hsa_circ_0000660	chr15:94899366-94945248:+	0.019867591	Down
hsa_circ_0007379	chr14:35020920-35024118:-	0.005379896	Down
hsa_circ_0123190	chr3:195415404-195435712:+	6.1567E-06	Down

*NA* not applicable. *P value < 0.05 was considered as statistically significant

### GO and KEGG analysis in LN

Gene Ontology (GO) analysis predicted the functions of differentially expressed circRNAs through the host genes based on three common aspects, including biological processes (BP), cellular components (CC), and molecular functions (MF). We selected the top 10 GO terms where circRNAs were significantly enriched in each of three biological functions. In the MF and CC category, we found that lysophosphatidic acid acyltransferase activity and apical plasma membrane were significantly regulated by the differentially expressed circRNAs in the LN kidneys. Moreover, in the BP category, enriched terms included biological regulation, cellular process, and metabolic process. Positive regulation of autophagy, which plays an important role in the pathogenesis of LN, was significantly regulated by these circRNAs in the BP category (Additional file 1: Supplement Figure 2).

Correspondingly, the Kyoto Encyclopedia of Genes and Genomes (KEGG) analysis can define the molecular function pathway of target host genes of the 159 dysregulated circRNAs. Target genes of differentially expressed circRNAs were enriched in pathways, such as apelin signaling pathway and tumor necrosis factor (TNF) signaling pathway. The molecular function of the associated apelin signaling pathway involves in regulating the expression of beclin1 and LC3, which are both important factors in autophagy, one of well-known pathogenesis in LN [16]. TNF-α contributes to the development of T cells, B cells, and dendritic cells in pathogenesis of SLE [17]. Both pathways were associated with activation of PI3K, which is an important factor in the pathogenesis of LN (Additional file 1: Supplement Figure 2).

### Validation of the selected circRNAs in the renal tissues

Since females are predominantly susceptible to lupus than males, ten female patients with LN were enrolled in this study, except for males. The characteristics of all 10 LN patients are shown in Table 3. With age and gender match, five females with renal tumor were used as the control group of the normal kidneys. In LN patients, urinary analysis showed 24-h urinary total protein excretion and numbers of red blood cell, white blood cell, and serology displayed levels of complements, erythrocyte sedimentation rate, serum creatine, albumin and routine blood indexes, the increased score of systemic lupus erythematous activity of disease indices (SLEDAI), and different severity of renal dysfunction. Ten LN patients presented various pathological classifications (class III, *n* = 2; class IV, *n* = 5; class III + V, *n* = 1; class IV + V, *n* = 2), when activity index (AI) and chronicity index (CI) were both assessed.

**Table 3:** Clinical characteristics of LN patients

			Serum							
Pt	Age (years)	Disease duration (months)	C3 (g/L)	C4 (g/L)	ESR (mm/h)	CRP (mg/L)	WBC (*10^9/L)	RBC (*10^12/L)	Hb (g/L)	PLT (*10^9/L)
1	39	144	0.43	0.06	37	3.06	2.80	3.09	89.0	104
2	38	48	0.65	0.11	27	3.13	3.64	4.15	116.8	209
3	34	19	0.59	0.10	60	54.83	6.40	2.69	81.0	96
4	20	36	0.19	0.07	3	3.23	2.32	3.90	115.9	128
5	23	6	1.03	0.15	12	3.13	5.56	5.46	117.8	221
6	35	36	0.25	0.07	38	3.13	2.13	3.30	103.3	106
7	19	60	0.56	0.11	10	3.13	4.60	2.37	74.0	110
8	48	156	0.34	0.08	14	1.00	5.40	2.84	85.0	80
9	36	1	0.33	0.05	11	9.00	2.10	3.45	105.0	136
10	46	120	0.62	0.05	74	32.52	3.50	3.14	98.0	227
	Serum			Urine			Renal biopsy			SLEDAI (score)
Pt	Creatinine (umol/L)	Urea (mmol/L)	eGFR (mL/min /1.73m^2^)	UTP (g/24 h)	RBC (/uL)	WBC (/uL)	Classification	AI (score)	CI (score)	
1	59	5.03	111.223	8.30	95	56	IV-S(A) + V	9	0	9
2	63	4.50	107.899	2.52	26	4	III-(A)	8	1	8
3	130	12.44	46.224	6.03	252	94	IV-G(A) + V	19	3	11
4	51	3.85	133.345	2.07	50	0	III-(A/C)	6	1	12
5	58	7.11	109.314	2.03	50	0	III-(A) + V	0	0	6
6	135	12.40	38.299	6.25	92	22	IV-G(A/C)	10	2	4
7	77	4.90	96.472	2.82	350	12	IV-S(A)	9	0	8
8	56	10.36	98.682	6.39	45	22	IV-G(A)	15	0	5
9	69	4.31	98.028	2.35	184	50	IV-S(A)	9	0	4
10	59	6.91	93.006	1.40	37	28	IV-S(A)	7	0	4

*Pt* patient, *WBC* white blood cells, *RBC* red blood cells, *Hb* hemoglobin, *PLT* Platelet, *C3* complement 3, *C4* complement 4, *AI* activity index, *CI* chronicity index, *eGFR* estimated glomerular filtration rate based on CKD-EPI formula, *SLEDAI* systemic lupus erythematosus disease activity index, *UTP* urinary total proteinuria per day

**Fig. 1:**
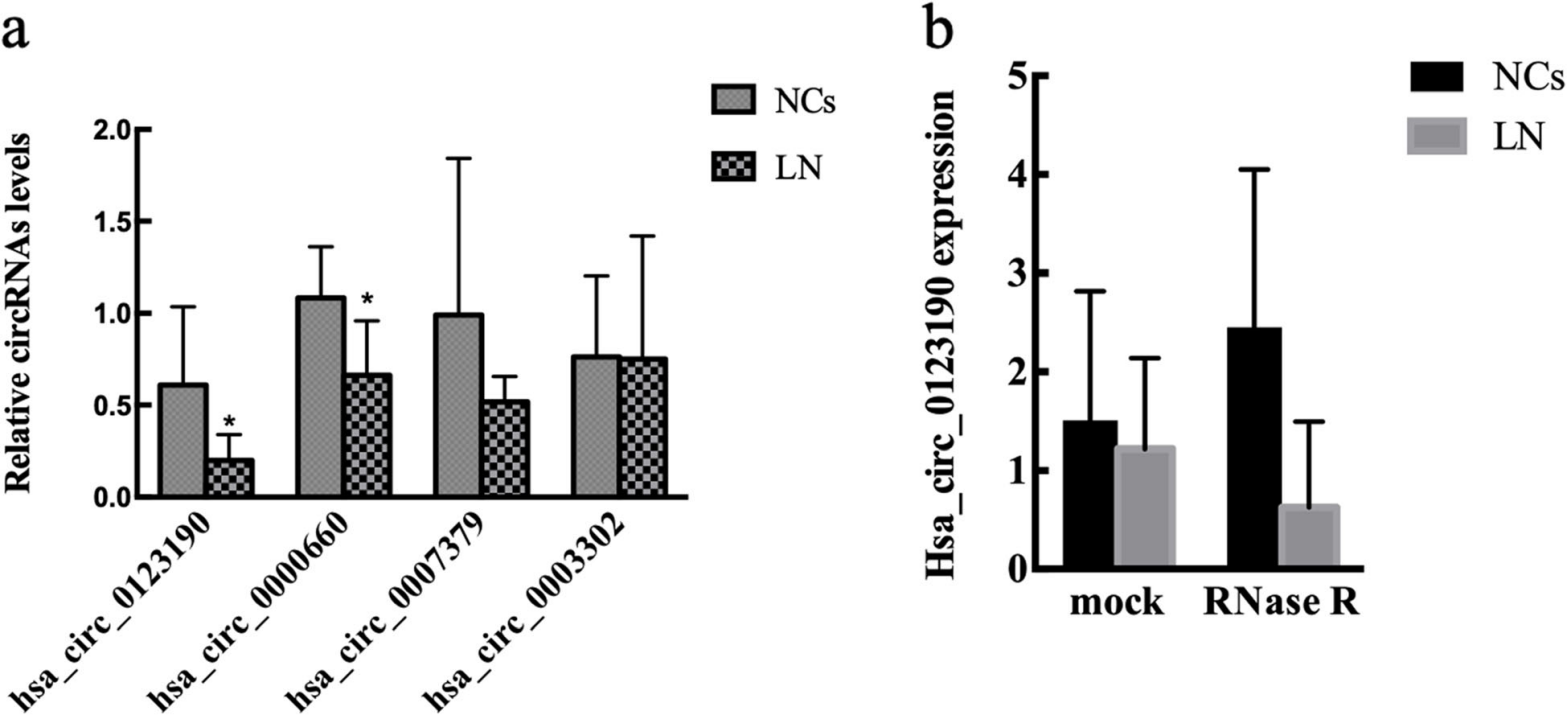
The expression of selected circRNAs in renal tissues. **a** The renal levels of circRNA from qRT-PCR were expressed as a ratio of average 2^−ΔΔCt^, of which each circRNA forms LN to NC. Hsa_circ_0123190 was significantly decreased in renal tissues. **b** Hsa_circ_0123190 was resistant to RNase R digestion. There was no remarkable difference in the expression of hsa_circ_0123190 before and after RNase R digestion (*P* > 0.05). Data are expressed as means ± SD (*P < 0.05)

We selected three downregulated circRNAs (hsa_circ_0000660, hsa_circ_0007379, and hsa_circ_0123190) and one upregulated circRNAs (hsa_circ_0003302) for the further validation in renal samples of 10 LN patients and 5 NC subjects by qRT-PCR. All four circRNAs showed similar expression patterns between the qRT-PCR and the circRNA sequencing. However, only hsa_circ_0123190 was significantly dysregulated between the renal tissues of patients with LN and NCs (*P* = 0.014) (Fig. 1a). Therefore, hsa_circ_0123190 was considered as a candidate for further analysis. Moreover, hsa_circ_0123190 was resistant to RNase R digestion (*P* > 0.05), suggesting that hsa_circ_0123190 is stable (Fig. 1b). The clinical significance of hsa_circ_0123190 in LN patients was investigated. There was no association of hsa_circ_0123190 with clinical parameters which we collected (all *P* > 0.05).

### Hsa_circ_012319 serves as a sponge for hsa-miR-483-3p

**Fig. 2:**
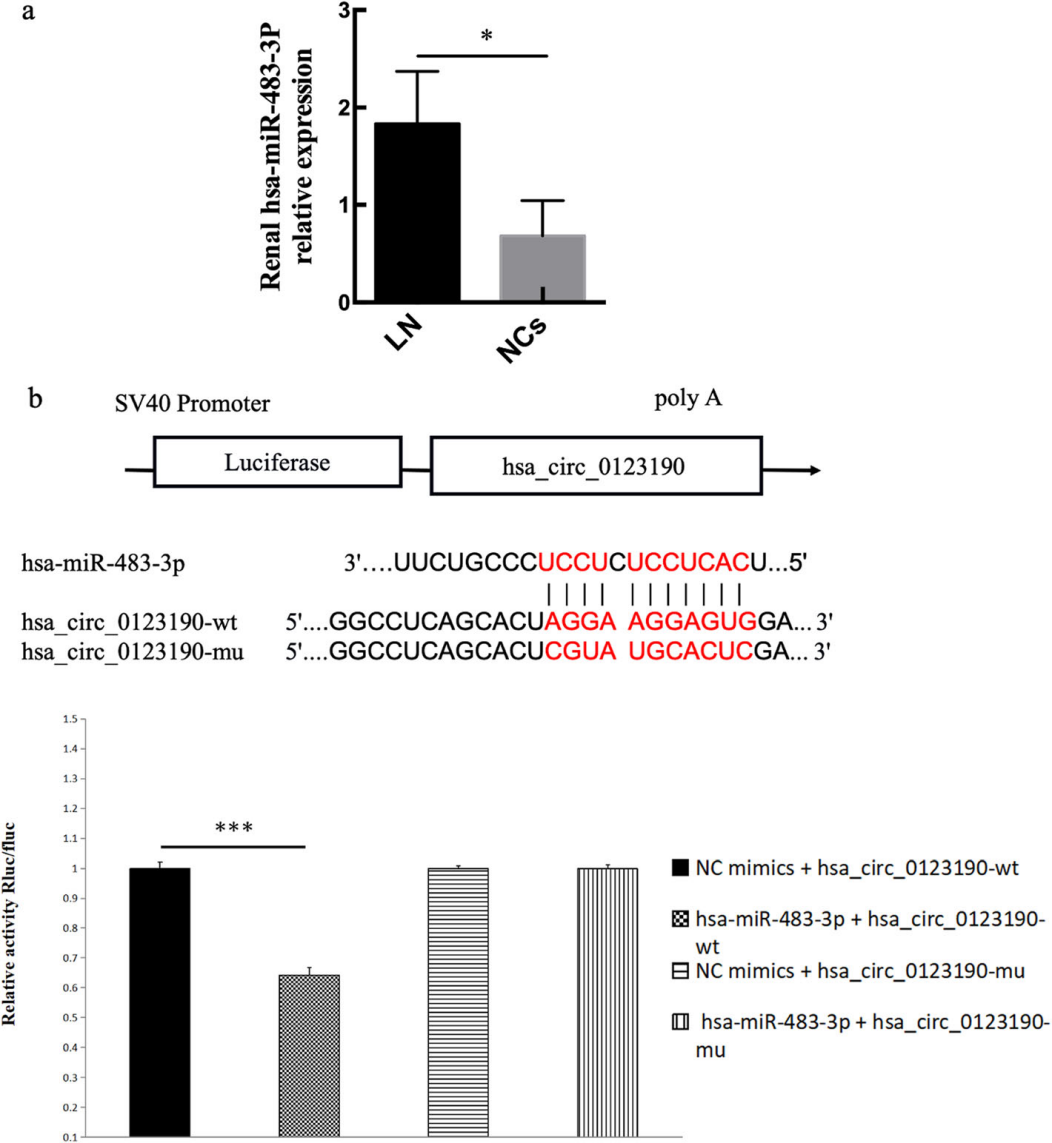
The interaction of renal hsa_circ_0123190 and hsa-miR-483-3p in LN. **a** The expression of hsa-miR-483-3p in the renal tissues was significantly increased in LN patients compared with controls. **b** Binding sequence prediction of hsa_circ_0123190 WT and hsa-miR-483-3p and sequence construction of hsa_circ_0123190 MUT. Dual-luciferase reporter assay showed the binding relationship between hsa_circ_0123190 and hsa-miR-483-3p in HEK293T cells

In view of the fact that circRNA can act as a miRNA sponge, the potential targets miRNAs of hsa_circ_0123190 were predicted using bioinformatics (CircInteractome, and miRanda). As a result, we found that hsa-miR-483-3p was the most likely complementary miRNA, which had a perfect match sequence to bind hsa_circ_0123190. We examined the expression of hsa-miR-483-3p in renal tissues using qRT-PCR. The results revealed that the expression of hsa-miR-483-3p was significantly increased in LN compared with NCs (*P* = 0.0498) (Fig. 2a). To further verify the hypothesis that hsa-miR-483-3p directly targets hsa_circ_0123190, we performed the dual-luciferase reporter assay with HEK293T cells, and the results showed that the relative luciferase activity was significantly reduced in cells co-transfected with hsa_circ_0123190 WT and hsa-miR-483-3p mimics compared with control. The relative luciferase activity was unchanged in co-transfected cell with hsa_circ_0123190 MUT and hsa-miR-483-3p mimics compared with control group (Fig. 2b). In a word, all results indicate that hsa_circ_0123190 targets hsa-miR-483-3p.

### APLNR is the target gene of hsa-miR-483-3p

**Fig. 3:**
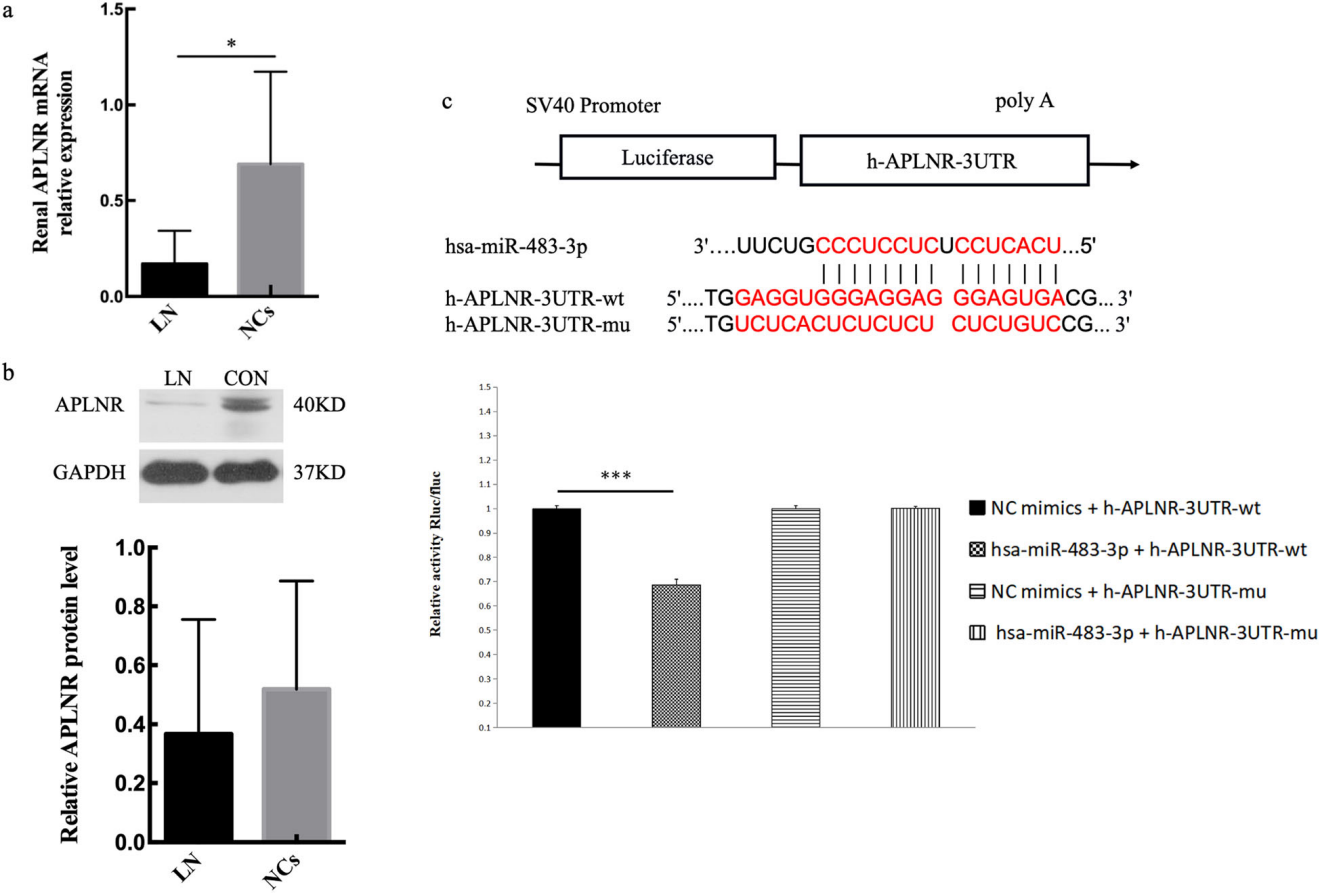
The expression of APLNR and the interaction of hsa-miR-483-3p and APLNR in LN. **a** The level of APLNR mRNA in the renal tissues was decreased in LN patients. **b** Western blot results showed the protein level of APLNR. **c** Binding sequence prediction of APLNR WT and hsa-miR-483-3p and sequence construction of APLNR MUT. Dual-luciferase reporter assay to validate the binding relationship between APLNR and hsa-miR-483-3p in HEK293T cells

The potential targets genes of hsa-miR-483-3p were searched with bioinformatics (targetScan and miRanda). Based on the NGS profiling of mRNA in this study, we found APLNR had perfect binding relationship with hsa-miR-483-3p. The expression of APLNR in kidneys was tested using qRT-PCR and Western blot (Fig. 3a, b). It was found that the mRNA level of APLNR showed a remarkable downregulation in the LN renal tissues compared with NCs. However, the APLNR protein expression was slightly decreased with no significance in the kidney of LN (*n* = 3) (*P* > .0.05). To further determine the interaction between hsa-miR-483-3p and APLNR, we transfected plasmids of h-APLNR-3UTR-WT and h-APLNR-3UTR-MUT into HEK293T cells. The luciferase activity is significantly decreased in the cells co-transfected with h-APLNR-3UTR-WT and hsa-miR-483-3p mimics compared with the control group. But the luciferase activity in co-transfected cells with h-APLNR-3UTR-MUT and hsa-miR-483-3p mimics had no significant difference compared with the control group (Fig. 3c). In addition, the level of APLNR in the peripheral blood was detected but failed.

### Renal fibrosis may correlate with the expression of APLNR

**Fig. 4:**
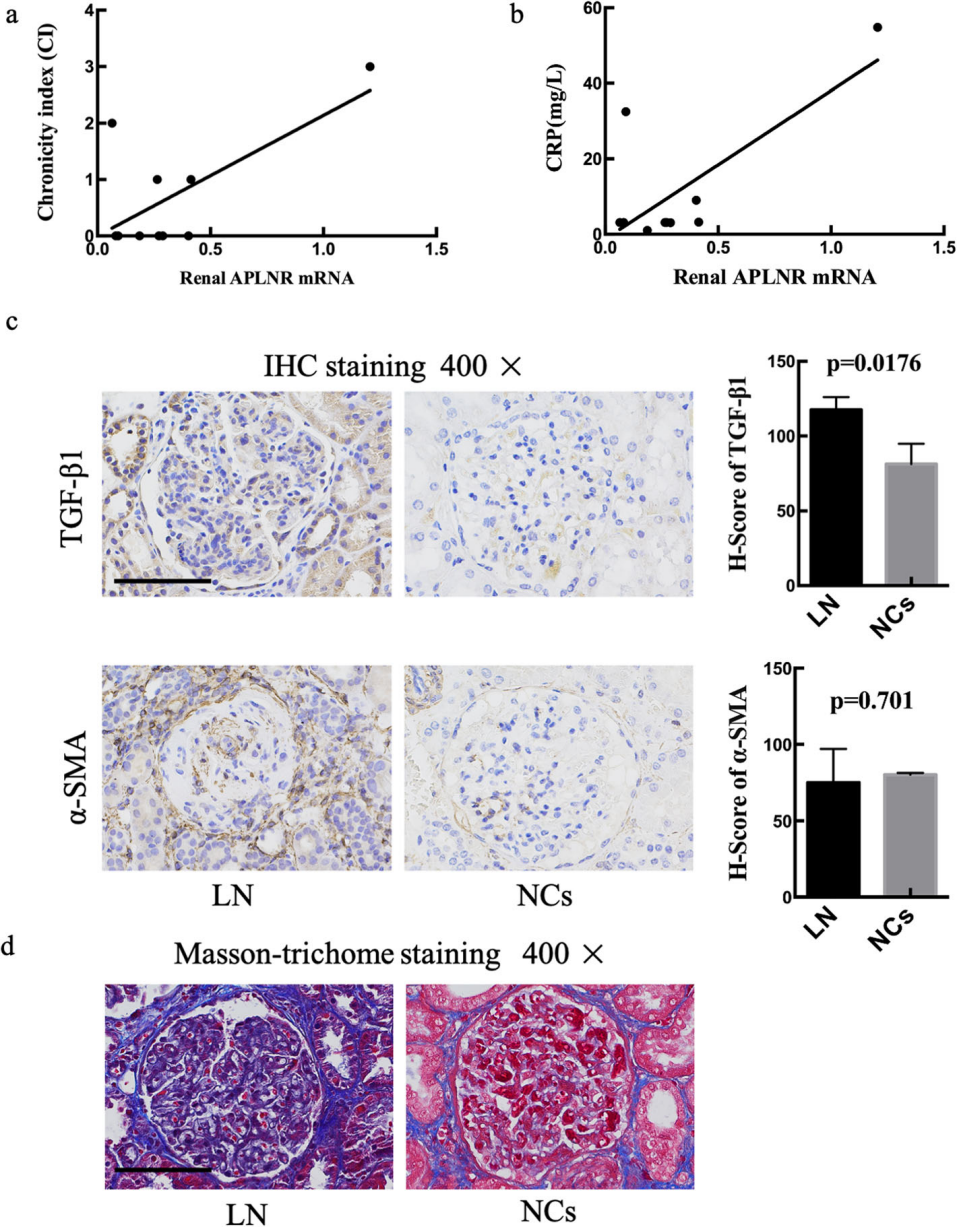
The level of APLNR associated with renal fibrosis in LN. The expression of APLNR mRNA was positively relevant with CI (**a**) and CRP (**b**). **c** The immunohistochemistry staining of fibrotic-related proteins including TGF-β1 and α-SMA, as well as the H-score of each protein. **d** Masson-trichome staining of paraffin-embedded kidney sections taken from patient with LN and controls. Data are expressed as means ± SD. (*P < 0.05, ×400 magnification, bar = 30 μm)

The relationship between the mRNA level of APLNR and the clinical characteristics of LN patients was explored. The results showed that the expression of APLNR was positively relevant with chronicity index (CI) (*P* = 0.033, *R*^2^ = 0.45) and C-reactive protein (CRP) (*P* = 0.015, *R*^2^ = 0.54) (Fig. 4a, b). APLNR, as apelin receptor, was involved in renal fibrosis by regulating the expression of TGF-β1 [13]. Pro-fibrotic protein TGF-β1 and α-SMA are central features of renal fibrosis [18]. Therefore, we assessed the extent of TGF-β1 and α-SMA IHC staining using H-score to semi-quantify. LN renal tissues showed significant high expression of TGF-β1 (*P* = 0.0176) compared with NCs (Fig. 4c). In addition, Masson-trichome staining was further demonstrated that fibrosis was significantly promoted in the LN group (Fig. 4d).

### Validation of hsa_circ_0123190 expression in peripheral blood of LN patients

**Fig. 5:**
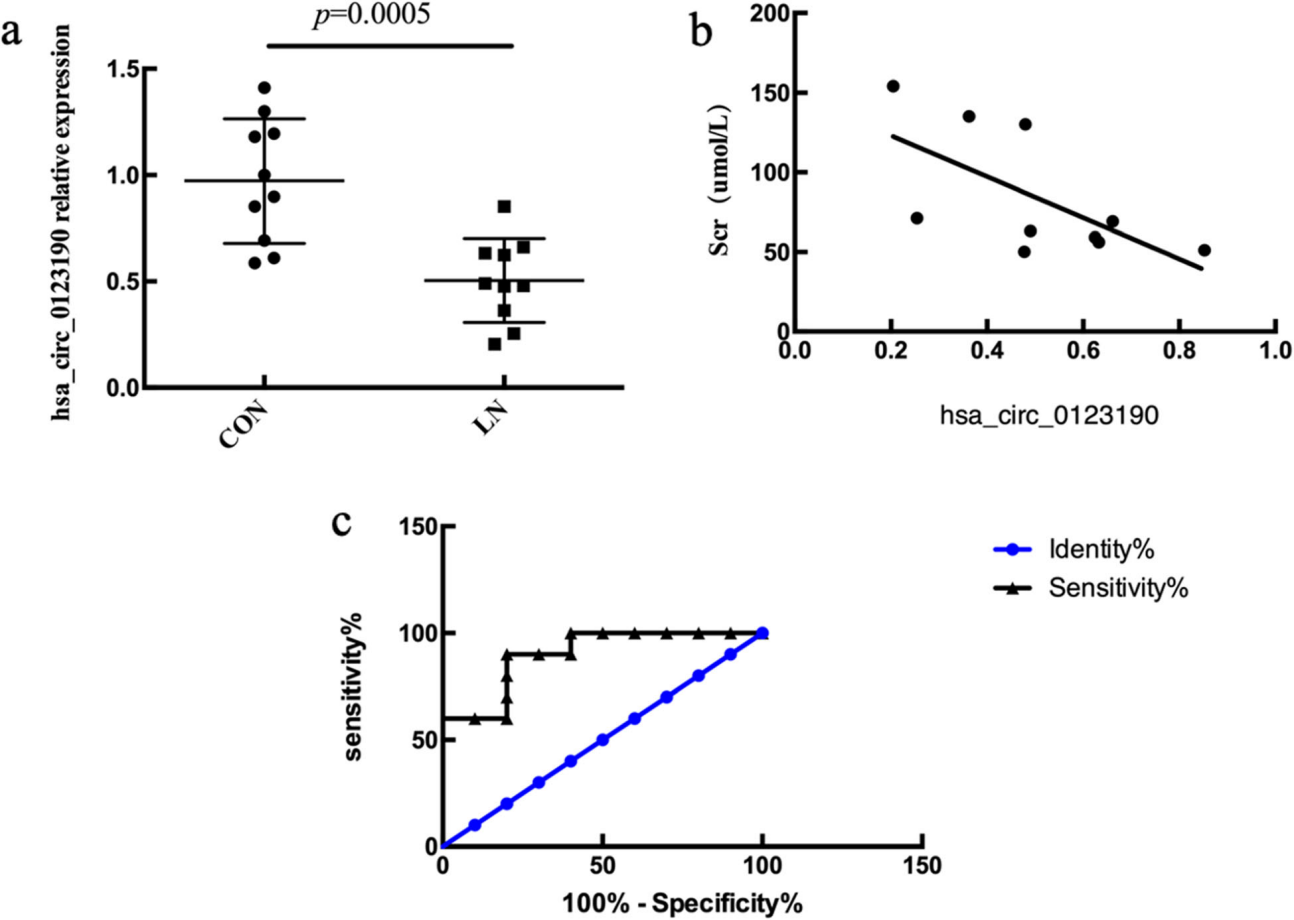
The expression and clinical relevance of hsa_circ_0123190 in peripheral blood of LN compared with NC. The levels of hsa_circ_0123190 from qRT-PCR were expressed as a ratio of average 2^−ΔΔCt^. Data are expressed as means ± SD (*P < 0.05). **a** Hsa_circ_0123190 was significantly decreased in peripheral blood samples. **b** Hsa_circ_0123190 was negatively associated with serum creatine levels. **c** The receiver operating characteristic (ROC) curve analysis of hsa_circ_0123190

The recent studies revealed that circRNA had tissue-specific manner, we identified hsa_circ_0123190 from the renal tissues. However, renal biopsy is an invasive and expensive operation, and hsa_circ_0123190 was supposed to be validated by qRT-PCR in peripheral blood of ten LN patients and ten healthy controls. As a result, the expression of hsa_circ_0123190 was remarkably decreased in LN patients (*P* = 0.0005) (Fig. 5a). In addition, we assessed the correlation between circRNA levels and clinical characteristics in LN patients. The expression of hsa_circ_0123190 was negatively correlated with leukocyte levels (*P* = 0.0123, *R*^2^ = 0.5638) and complement 4 (C4) levels (*P* = 0.0099, *R*^2^ = 0.5855). Moreover, hsa_circ_0123190 expression was significantly associated with serum creatine levels (*P* = 0.044, *R*^2^ = 0.4151) (Fig. 5b).

The important utility of hsa_circ_0123190 was further explored by ROC curve analysis. The area under the curve (AUC) for hsa_circ_0123190 in the peripheral blood when distinguishing LN patients from NCs was 0.900 (95% CI 0.7659–1.034, *P* = 0.0025). The maximum Youden’s J index (sensitivity and specificity) was 70% (90% and 80%) for hsa_circ_0123190 to differentiate LN patients from controls, and the corresponding optimal cutoff values was 0.6773 (Fig. 5c).

## Discussion

The poor prognosis of LN is still a seriously clinical and economic problem, as the mechanisms of LN remain indistinct [19]. Thus, the identification of novel treatment targets for LN would be very desirable. CircRNA is a special class of endogenous RNAs with multiple functions [20]. Many studies have revealed the abnormal circRNAs were associated with several renal diseases, such as AKI [21] and carcinoma [8, 22]. However, the role of circRNA in LN has been rarely reported. In the present study, we conducted integrative analysis using circRNA sequencing in renal tissues and identified 159 circRNAs with significantly differential expression, of which 73 were upregulated and 86 downregulated. Hsa_circ_0123190 maybe act as a sponge for hsa-miR-483-3p, which regulates APLNR expression in LN. In addition, peripheral blood hsa_circ_0123190 would be a biomarker for patients with LN.

Special attention must be paid to the type of specimen since circRNAs were highly expressed in a cell type-specific or tissue-specific manner [4, 5]. Ouyang et al. discovered that upregulated plasma circRNA_002453 level in LN patients was associated with the severity of renal involvement and served as a novel biomarker for LN patient diagnosis [23]. Luan et al. showed that circHLA-C played an important role in the pathogenesis by sponging miR-150 in LN [24]. These data are not consistent across researches due to different samples and methods. For instance, Luan et al. reported a circRNA profiling in single class IV of LN and found seven differentially expressed circRNAs. The reasons why the results were different from our study may be as follows: different sample sources, different sample sizes, and different renal pathological types. Compared with study by Luan et al., the present study covered the common proliferative classification of LN to explore the role of circRNA and validated differentially expressed circRNAs both in the peripheral blood and kidney tissues. In addition, we performed ROC curve analysis to ensure the clinical value of the circRNAs as diagnostic biomarkers for LN. The profiling of circRNAs from this study may provide a novel database and new view to study mechanisms of LN.

Over the last few years, many functions of circRNAs have been elucidated. CircRNAs can act as gene expression regulators via different regulatory modes [25], such as “sponges” miRNAs resulting in the expression of target mRNAs [7]. The interaction network of circRNA in LN was slightly shed in the current study. The circRNA hsa_circ_0123190 was downregulated in the renal tissues of patients with LN, and the expression of hsa-miR-483-3p had a significantly opposite direction. Bioinformatics analysis and luciferase reporter assay discovered that hsa_circ_0123190 directly bind to hsa-miR-483-3p, which serves as a miRNA sponge. There have been several reports that increased hsa-miR-483-3p impair endothelial cell survival to limit vascular repair capacity upon injury in cancer and metabolic diseases [26–28]. However, the role of hsa-miR-483-3p has been unreported in LN, which also has vascular lesion. This suggests that the downregulated hsa_circ_0123190 might sponge hsa-miR-483-3p in LN to promote kidney vascular damage.

In addition, hsa-miR-483-3p was only interacted with APLNR in this study, which was decreased in the renal tissues of LN. APLNR is the orphan G protein-coupled apelin receptor, which could be expressed in various organ and tissues [13]. Hus-Citharel et al. discovered that the APLNR mRNA was widely expressed in the rat kidney, and the level of APLNR in all nephron segments was lower than the glomeruli [29]. There are multiple functions of APLNR and apelin, such as regulation of blood pressure, immune response, and anti-inflammatory effect [30]. They also play an important role in organ fibrosis [14]. Renal fibrosis is a major feature of chronic kidney disease including LN. Collagen, α-SMA, and TGF-β are considered to be important fibrosis-related proteins. In the present study, Masson-trichome staining laterally demonstrated that renal fibrosis is a typical pathological manifestation of the LN kidney. A large amount of studies on kidney diseases indicates that APLNR and apelin can improve renal interstitial fibrosis by restraining the expression of TGF-β1 [14]. TGF-β participates in chronic renal inflammation and renal fibrosis through the Smad signaling pathway, protein kinase C pathway, and mitogen-activated protein kinase pathway. A research on mice with complete unilateral ureteral obstruction (UUO) illustrated that apelin treatment could significantly reduce the expression of α-SMA, TGF-β1, and its receptor [31]. In this study, we found that the expression of TGF-β1 was significantly increased in LN, which was opposite to APLNR. Therefore, we tentatively hypothesized APLNR may be involved in renal fibrosis of LN by regulating TGF-β1.

The expression of peripheral blood hsa_circ_0123190 was downregulated and negatively associated with serum creatine level in LN, which indicated that it might be involved in renal injury in patients with LN. Moreover, we ensured the clinical value of this circRNA as a diagnostic biomarker for LN through performing ROC curve analysis. These results indicated that hsa_circ_0123190 in the peripheral blood could be a novel promising diagnostic and non-invasive biomarker of LN patients.

There are some limitations in our study. First of all, our sample sizes were comparatively small. The examination of circRNAs in the kidneys and blood samples in larger cohorts of LN patients may define its clinical value as a diagnostic biomarker. Secondly, the mechanisms of hsa_circ_0123190 in the development and pathogenesis of LN has not yet been completely studied. Thus, further experiments in vitro and in vivo are needed. Thirdly, the expression of hsa_circ_0123190 in T cells, B cells, and other immune cells will be detected and compared in the next step.

## Conclusion

**Fig. 6:**
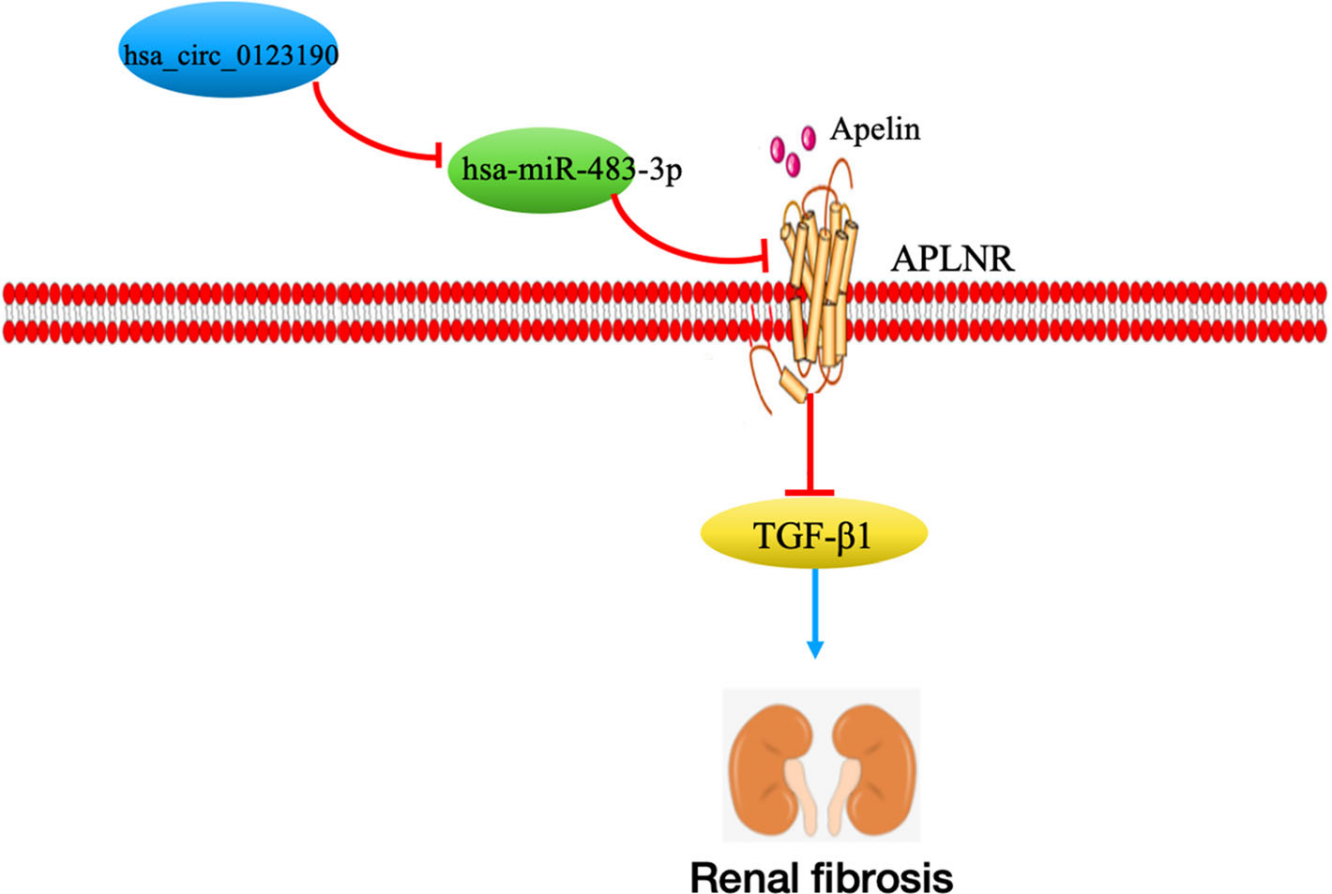
The role of hsa_circ_0123190 in LN. Hsa_circ_012319 can regulate APLNR expression by sponging hsa-miR-483-3p, and then, APLNR is involved in renal fibrosis by inhibiting TGF-β1

In a conclusion, the study provided a profile of renal circRNAs for patients with LN. Hsa_circ_0123190 can regulate APLNR expression involved in renal fibrosis by sponging hsa-miR-483-3p in LN (Fig. 6). Hsa_circ_0123190 in the peripheral blood was a non-invasive and novel biomarker for LN. Further work needs investigation of hsa_circ_0123190 in larger cohorts of LN patients and mechanisms of hsa_circ_0123190 in LN in vitro and in vivo experiments.

## Supplementary Information

**Additional file 1.** Figure S1. Profiling and characteristics of circRNAs in LN renal tissues. Figure S2. Go and KEGG analysis.

**Additional file 2.** Table S2. Clinical information of LN

**Additional file 3.** Table S1. Clinical information of NCs

## Data Availability

The datasets analyzed during the current study are available from the corresponding author on reasonable request.

## Abbreviations

LN: Lupus nephritis
SLE: Systemic lupus erythematosus
CircRNAs: Circular RNAs
NCs: Normal controls
APLNR: Apelin receptor
ceRNA: Competitive endogenous RNA
NGS: Next-generation sequencing
qRT-PCR: Quantitative reverse transcription polymerase chain reaction
IHC: Immunohistochemistry
CI: Chronicity index
AI: Activity index
TGF-β1: Transforming growth factor-β1
α-SMA: α-Smooth muscle
ESRD: End-stage renal disease
ROC: Receiver operating characteristic
AUC: Area under the curve
GO: Gene Ontology
BP: Biological processes
CC: Cellular components
MF: Molecular functions
KEGG: Kyoto Encyclopedia of Genes and Genomes
TNF: Tumor necrosis factor
CRP: C-reactive protein
C4: Complement 4
AKI: Acute kidney injury
FOXP4: Fork head box P4
