# Supplementary material for: Hsa_circ_0123190 acts as a competitive endogenous RNA to regulate APLNR expression by sponging hsa-miR-483-3p in lupus nephritis
Source: Arthritis Res Ther. 2021 Jan 13;23:24. doi: 10.1186/s13075-020-02404-8 (PMC7805051; doi:10.1186/s13075-020-02404-8)
Supplement: Supplementary file 1 — Additional file 1. Figure S1. Profiling and characteristics of circRNAs in LN renal tissues. Figure S2. Go and KEGG analysis. [file 13075_2020_2404_MOESM1_ESM.pdf]

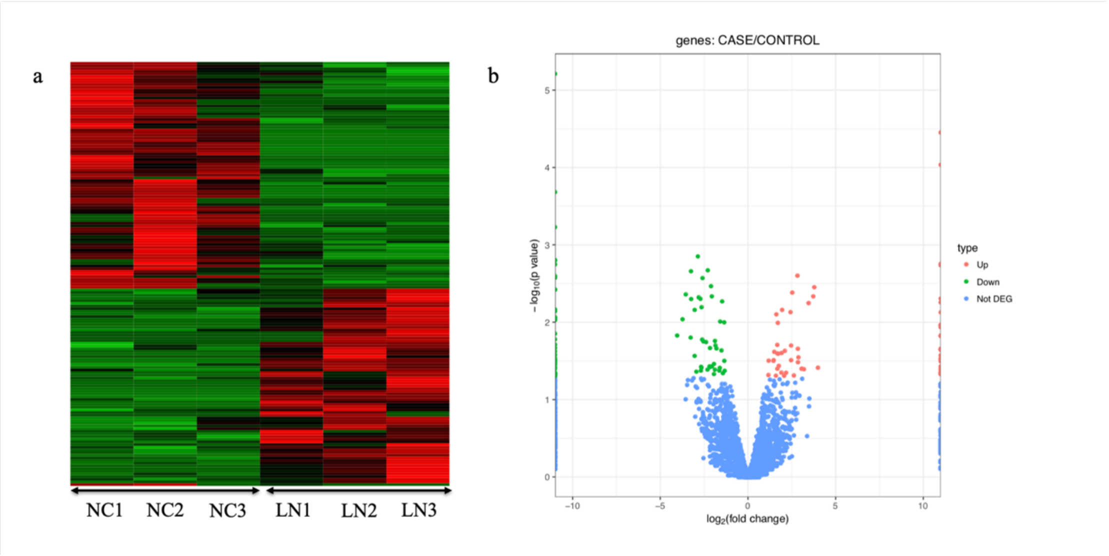

Supplement Figure 1. Profiling and Characteristics of circRNAs in LN Renal Tissues. (a) Clustered heatmap with each column representing a kidney tissue and each row representing a circular RNA identified by circRNA sequencing. The red indicates upregulated circRNAs, and the green indicates downregulated circRNAs. (b) Volcano plots showed abnormally expressed miRNAs between LN and normal controls. Red spots indicated the upregulated miRNAs, and blue spots indicated the downregulated miRNAs.

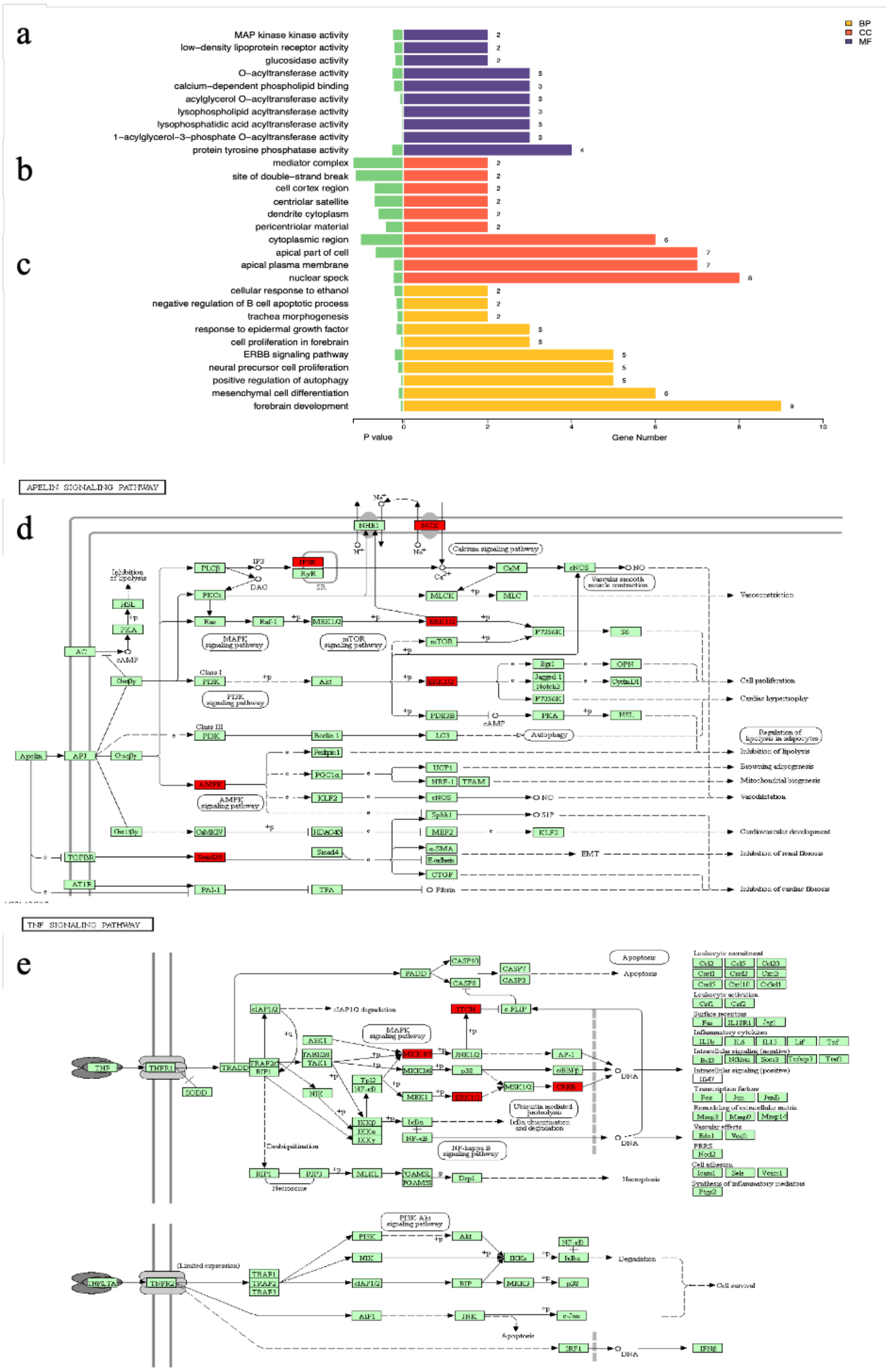

Supplement Figure 2. GO and KEGG analysis. The top 10 predicted functions of the host gene regulated by circRNAs in LN were obtained with GO analysis. They were categorized based on (a) MF, (b) CC and (c) BP. The arrows indicate functions significantly involved in the pathogenesis of LN. KEGG analysis showed 29 signal pathways relate to differentially regulated circRNAs in LN. (d) Apelin and (e) tumor necrosis factor (TNF) signal pathways were 2/29 pathways obtained from KEGG analysis
